# The Effects of Two Organic Soil Amendments, Biochar and Insect Frass Fertilizer, on Shoot Growth of Cereal Seedlings

**DOI:** 10.3390/plants12051071

**Published:** 2023-02-27

**Authors:** Aaron Carroll, Mark Fitzpatrick, Simon Hodge

**Affiliations:** School of Agriculture & Food Science, University College Dublin, D04 N2E5 Dublin, Ireland

**Keywords:** barley, black soldier fly, circular economy, *Hermetia illucens*, oats, spelt, triticale

## Abstract

To mitigate the environmental harm associated with high-input agriculture, arable farmers are increasingly required to maintain productivity while reducing inputs of synthetic fertilizers. Thus, a diverse range of organic products are now being investigated in terms of their value as alternative fertilizers and soil amendments. This study used a series of glasshouse trials to investigate the effects of an insect frass-based fertilizer derived from black soldier fly waste [HexaFrass™, Meath, Ireland] and biochar on four cereals grown in Ireland (barley, oats, triticale, spelt) as animal feed and for human consumption. In general, the application of low quantities of HexaFrass™ resulted in significant increases in shoot growth in all four cereal species, along with increased foliage concentrations of NPK and SPAD levels (a measure of chlorophyll density). These positive effects of HexaFrass™ on shoot growth were observed, however, only when a potting mix with low basal nutrients was used. Additionally, excessive application of HexaFrass™ resulted in reduced shoot growth and, in some cases, seedling mortality. The application of finely ground or crushed biochar produced from four different feedstocks (*Ulex*, *Juncus*, woodchip, olive stone) had no consistent positive or negative effects on cereal shoot growth. Overall, our results indicate that insect frass-based fertilizers have good potential in low-input, organic, or regenerative cereal production systems. Based on our results, biochar appears to have less potential as a plant growth promoting product, but could be used as a tool for lowering whole-farm carbon budgets by providing a simplistic means of storing carbon in farm soils.

## 1. Introduction

To meet growing food demands and to increase productivity, many cereal farmers have relied on high inputs of synthetic fertilizers in combination with the breeding of new high-yield plant lines. These conventional arable farming systems can be highly productive but are under increasing scrutiny because of the negative environmental impacts associated with synthetic fertilizers, pesticides, and deep ploughing. To produce food more sustainably, agronomists are now exploring alternative ways to maintain yields while decreasing whole-farm environmental footprints [1,2,3,4]. These initiatives often involve the incorporation of various organic amendments to improve soil fertility, promote a healthy soil biome, and enhance soil carbon content, principles now associated with organic and regenerative farming systems [5].

Two organically derived soil amendments currently undergoing substantial research in terms of their effects on plant performance are biochar and insect frass fertilizers. Biochar is a carbon-rich substance produced by the pyrolysis of biomass at relatively low temperatures (<700 °C), and can be produced intentionally for use as a soil amendment or as a by-product of biomass pyrolysis for bioenergy production [6,7]. The plant-enhancing properties of biochar have been reported for almost 200 years [8], although it was not until the last few decades that a sustained effort has been made to understand the mechanisms of how biochar influences plant growth and soil bio-physiochemistry [9,10,11,12]. A secondary research interest in biochar has arisen because of its potential to allow for long-term carbon sequestration in soils, and it therefore has potential to be a component of whole-farm carbon budgets [9,11,12,13,14].

Several mechanisms have been proposed as to how biochar could improve plant performance. Biochar has been shown to increase both the water holding capacity and water retention of soils, and thus mediate drought stress in some cases [15,16,17,18]. In addition to holding water, biochar has also been reported to increase nutrient retention within soils, which has both agronomic and environmental benefits [10,19,20]. Biochar can also have a liming effect, which can subsequently increase the utilization of some macro- and micro-nutrients [9,10,18,21,22]. 

The European Union has recently authorized the use of farmed insects as feed for pigs, poultry, and aquaculture, which has created new markets for insect feed production. This industrial-scale insect farming produces substantial quantities of waste, generally known as ‘frass’, that contains insect feces, exuviae, fragments of exoskeleton, diet material, and microorganisms [23,24]. This insect frass can be repurposed as an organic fertilizer, and there is a growing body of evidence indicating that this insect frass fertilizer (IFF) is capable of producing yields equivalent to those obtained when applying synthetic N fertilizers and more traditional organic fertilizers [25,26,27,28]. For example, Choi et al. (2009) reported that the growth and nutrient composition of cabbages treated with a commercial N fertilizer and IFF were almost identical [29], and, similarly, Quilliam et al. (2020) found that shallots and maize grown with an NPK fertilizer, chicken manure, or IFF had no significant differences in yield [30]. Tanga et al. (2022) reported that the application of IFF led to a significant increase in maize grain yield by 153% when compared with that obtained after the application of a commercial organic fertilizer [31]. 

As biochar and IFF represent commodities derived from waste products, they provide excellent opportunities for the practical implementation of circular economy models [32]. Biochar, for instance, can provide an alternative use for agricultural waste products, such as rice hulls, scrub clearance, and wood clippings, that may otherwise be left unused [10]. IFF is also primarily produced from organic waste products, such as brewers spent grain, municipal green waste, food waste, animal manures, and sewage sludge, which may otherwise be dumped in landfill sites or incur expensive processing costs [33]. The potential benefits to plant performance from combining applications of biochar and IFF have already received some attention. For example, Tan et al. (2021) described substantial positive effects of incorporating a soldier fly-derived IFF in a biochar-based growing medium for the production of leafy vegetables such as pak choi [34]. In other cases, the effects of adding biochar along with IFF are not so clear. For example, Hodge and Conway (2022) reported that only some combinations of biochar and IFF resulted in enhanced growth of chicory and plantain compared with IFF alone [35], and Butnan et al. (2022) described dose-specific and temporally inconsistent effects of combining eucalyptus biochar and cricket frass fertilizer on the yield of kale [36]. Although consistent benefits of IFF/biochar mixtures have not yet been demonstrated, commercial products combining biochar and IFF are already available on the market (e.g., GreenMan Char, greenmanchar.com.au/products/biochar-frass).

As research into the potential of IFF develops, several recent investigations have examined the responses of cereals such as barley, rice, and maize to the application of IFF derived from different insect species. For example, Houben et al. (2020) found that the IFF derived from mealworms increased the biomass of barley to the same extent as mineral fertilizers [37], whereas Gebremikael et al. (2022) found that IFF derived from black soldier flies had a positive effect on maize, but this was dependent upon the food waste on which the flies had been reared [38]. Several studies have also been performed investigating the effects of biochar on the growth of cereals, although these often report inconsistent or no benefits to the target plants. For example, Jones et al. (2012), in a three-year study in Wales, found that biochar had no effect on the yield of maize [21], and Gathorne-Hardy et al. (2009) reported that biochar had no significant impact on the yield of spring barley in the UK [39]. Laird et al. (2017) found that biochar had no effect on the aboveground biomass of corn at most of their study sites, but they did observe increased crop biomass at one location with poor soils [40].

Around 300,000 ha of cereal crops are grown in Ireland each year, with some of the most common being spring and winter oats (~14,000 ha each), winter barley (~67,000 ha each), and spring barley (~142,000 ha each) (www.cso.ie; accessed on 6 December 2022). Spring barley and spring oats are mainly used as animal feed, although some spring oats are kept for human consumption and a small amount of barley (~13%) is used for malting for beer and whiskey production. Similarly, almost all winter barley and winter oats are used for animal feed, with a proportion of winter oats used for milling or rolling for porridge (Teagasc. www.teagasc.ie/crops/crops/cereal-crops/, accessed on 6 December 2022). On a smaller scale, because of the high proportion of Irish cereals that are processed as animal feeds, another cereal under investigation is triticale, which is a cross between rye and wheat, and has potential to produce higher forage and grain yields compared with winter wheat under typical Irish growing conditions [41]. In terms of bespoke crops that are increasing in popularity in Ireland, one of the main cereals is spelt (*Triticum spelta*), which has been grown in Ireland since the Iron Age [42]. Spelt is favored by some artisan bakers because its flour has high protein and high fiber content and has a more digestible gluten structure compared to wheat flour. 

As new IFF products enter the market and novel biochars are created from innovative feedstocks, there is a need to assess their efficacy on a range of plant species, at different application rates, and under different growing conditions. The primary objective of this study, therefore, was to evaluate the effects of different biochars and HexaFrass^TM^ (HF), a novel IFF produced in Ireland, on several cereals of Irish relevance. Using greenhouse trials, we examined how seedling shoot dry weight of four cereals (oats, barley, spelt, triticale) was affected by the application of both biochar and HF applied alone and in combination. We also examined the effects of different forms of biochar (feedstock, particle size) and compared the response in shoot growth obtained using HF with that obtained using a standard organic fertilizer (chicken manure). To assess the effects of HF and biochar on foliage quality, we used a SPAD meter to provide a measure of leaf chlorophyll content and obtained measurements of foliage macro- (NPK) and micro-nutrients (Mg, Ca).

## 2. Materials and Methods

### 2.1. General Methods

#### 2.1.1. Fertilizers and Biochar

HexaFrass^TM^ [HF; Hexafly, Co. Meath, Ireland] is a commercially available fertilizer produced by rearing black soldier fly (BSF; *Hermetia illucens*) larvae on brewery waste [24]. HF typically contains 60% organic matter, is rich in chitin, and has an N–P–K ratio of approximately 3:2:1. Nutrient analysis has shown that HF also contains important plant micronutrients, such as sulphur (6 g/kg), magnesium (5 g/kg), iron (300 mg/kg), and copper (12 mg/kg) [24]. The pH of an aqueous 1:1 HF solution was found to be approximately neutral at 7.3. To compare the effects of HF with a standard fertilizer, Westland Organic Chicken Manure Pellets (chicken manure; CM) were also used in one trial (see below). This organically certified fertilizer has an N–P–K ratio of 4.5–3.5–2.5 and has been used in previous studies as a positive control treatment for comparison with HF [24,35].

All biochars were manufactured by Argina Fuels, Roscommon, Ireland, and supplied by Biochar Ireland, Lough Derg, Ireland. The trials in this investigation used biochars derived from four different feedstocks: hardwood and olive stones; rushes (*Juncus* spp); spruce wood chips (*Picea sitchensis*); and furze (*Ulex* spp). The olive stone and *Ulex* feedstocks were pyrolyzed using a Kon-Tiki cone at approximately 500 °C, whereas the biochar from the rushes and spruce wood chips were created at lower temperatures of around 400 °C. Because a quenching process was used that resulted in high water content of the final product (up to 50% by weight), the biochar was first dried at 105 °C for 3 days. The resulting dry biochar was then either ground to a fine powder using an electronic grinder or crushed and passed through a pair of sieves to obtain a fraction consisting of particles between 3 mm and 7 mm in size.

Fertilizer and biochar treatments were added to growing media prior to the introduction of seedlings. Application rates were weighed and added to pots individually, and then mixed thoroughly with the growing medium using a metal spoon.

#### 2.1.2. Potting Mixes

Most trials used a potting mix consisting of equal parts by volume of Westland Nutrient Rich Garden Soil, Plagron Coco Bric coir fiber, and vermiculite. The coir fiber and vermiculite contain negligible quantities of nutrients, and although the garden soil is high in humus and ‘natural slow-release nutrients’, since it only made up a third by volume this growing medium was designated as a ‘low nutrient’ potting mix. Chemical analysis [Southern Science Laboratories, Kerry, Ireland] of this potting mix indicated N–P–K values of 0.3–0.02–0.5 by dry weight.

In one trial, a contrasting ‘high nutrient’ potting mix was created by mixing Westland Multi-Purpose Compost in a ratio of 2:1:1 by volume with the Westland Nutrient Rich Garden Soil and vermiculite. The multi-purpose compost is very high in organic matter (70% peat) and produces a richer, more nutrient-dense, growing medium. Chemical analysis of this potting mix indicated N–P–K values of 1.7–0.3–0.3 by dry weight content. As confirmation of the difference in nutrient content between the two types of growing media, the shoot dry weight of the plants grown in the high nutrient mix was over twice that in the low nutrient growing medium.

#### 2.1.3. Test Plants, Growing Conditions, and Harvesting

Four cereals were used in these trials: oats (*Avena sativa* cv. Apollon), spelt (*Triticum spelta*), triticale (*Triticosecale* cv *Trisem*) and barley (*Hordeum vulgare* cvs. Westminster and Quadriga). All seeds were sourced from Fruit Hill Farm, Co. Cork, Ireland. Seeds were germinated inside plastic containers on wet paper towels in a dark cupboard at room temperature for five days. Seedlings were then transplanted to individual pots (7 × 7 cm), which were then placed onto individual metal dishes to prevent leaching of nutrients and cross contamination of fertilizer treatments. 

Trials were conducted in greenhouses at Rosemount Environmental Station, University College Dublin (53.305349, −6.233302), between February and August 2022. During this period, the greenhouse had an average daily temperature of approximately 18 °C (range 15–33 °C) and an average daily relative humidity of 51% (range 42–83%). No artificial lights were used throughout the trials. When required, all plants were treated with a pyrethrin-based aphicide (Bugclear Ultra Gun™) and the fungicide Talius^®^ to control powdery mildew. Experimental treatments were arranged on the greenhouse benches using a complete randomized block design in all trials. 

Plants were watered with untreated water every 2 to 4 days, and were always watered the day prior to harvesting. Plants were harvested 6 to 7 weeks after the seedlings were transplanted. At harvest, the shoots and foliage (‘shoot’) were cut at the soil surface, and, in some trials, shoot fresh weight (fwt) was then obtained. All shoots were placed in paper bags, dried in an oven at 65 °C for 3 days, and then the dried shoot weight (dwt; mg) obtained using an electronic balance (Bonvoisin Electronic Analytical Balance). On occasion, shoot dry matter content (DM; %) was calculated as 100 × (dwt/fwt).

A summary of the various trials investigating the effects of HexaFrass and biochar on cereal shoot growth and foliage chemistry is provided in Supplementary Table S1.

### 2.2. The effect of HexaFrass™ on Cereal Shoot Growth: Application Rate and Basal Nutrient Conditions

To examine how the growth of barley (cv Quadriga), oats, spelt, and triticale responded to increasing amounts of frass fertilizer, HF was applied at the following rates to the standard low nutrient potting medium: 0, 1, 2, 4, 8, 12, 16 g per pot. For oats, there were ten replicates per application rate, whereas for the remaining three cereals, there were eight replicates for most application rates and four replicates for the 12 g and 16 g application rates. 

To examine whether any positive effects of HF on plant performance were mediated by the basal nutrient levels of the potting media, additional barley and oat plants were grown in the high nutrient potting medium (described above), with the addition of 0 g HF or 4 g HF. To establish whether HF could achieve similar effects on shoot growth compared with a standard organically certified fertilizer, based on protocols used in previous investigations [24,35] we grew additional plants in both the high and low nutrient potting media with the addition of 2 g of powdered chicken manure (CM). Each potting media/fertilizer treatment combination was replicated ten times with each plant species.

### 2.3. Response of Four Cereals to a Combination of HexaFrass™ and Biochar 

It was desirable to investigate whether different cereals showed consistent responses to the addition of HF and biochar, and whether there were synergistic effects when the two products were applied in combination. Therefore, seedlings of barley (cv Quadriga), oats, spelt, and triticale were grown in the low nutrient potting media with the addition of HF (0 g or 3 g) and powdered olive stone biochar (0 g or 2 g). A factorial experimental design was used, and all plant/HF/biochar combinations were replicated eight times.

### 2.4. Response of Barley to Four Types of Biochar in Granular and Powdered Forms

To examine how biochar feedstock and form might affect shoot growth of cereals, a factorial experiment was used with barley (cv Quadriga) as the test plant. Biochar from the four different feedstocks (*Ulex*, *Juncus*, olive stones/hardwood, and spruce wood chips) was added to pots containing the low nutrient potting mix at three rates (0 g, 2 g, or 4 g per pot) and in two forms, powdered or crushed, with grains between 3–7 mm. These biochar treatments were combined with the addition of HF at two rates (0 g and 3 g per pot), which produced 34 treatments in total [((4 biochar feedstocks × 2 biochar quantities × 2 biochar forms) + No biochar) × 2 HF rates]. Each treatment was replicated six times with the exception of the overall control treatment (0 g biochar, 0 g HF), which was replicated 12 times. 

### 2.5. Response of Barley and Oats to Biochar Application Rate with and without HexaFrass™

To examine how the growth of oats and barley (cv Westminster) responded to increasing amounts of biochar, powdered olive stone biochar was added to the low nutrient potting mix at the rates of 0, 0.5, 1, 2, and 4 g per pot. In addition, HF was applied to pots at rates of 0 g and 2 g, creating ten treatments for each plant species, with each plant/treatment combination being replicated eight times. 

### 2.6. Qualitative Changes in Cereal Foliage Following Application of Biochar and HexaFrass™

To gain an indication of how foliage nutrient levels responded to biochar and HF, additional barley plants (cv Quadriga) were grown in larger (2 L) pots filled with the low nutrient compost. HF was added to pots once seedlings were established (~5 cm tall; one plant per pot) at rates of 0, 4, 8, and 12 g. Additionally, 8 g of powdered olive stone biochar was added to pots containing 0 g and 4 g HF. The plants were allowed to grow for 8 weeks and were then harvested. The fwt and dwt weight of each plant were obtained and then the dried shoots were ground to a fine powder. For the fwt and dwt measurements, eight replicate plants were set up for the controls and 4 g HF treatments, and six replicates for the other treatments. The dried ground shoot material from pairs of plants in the same treatment were then pooled for chemical analysis. This material was analyzed by a commercial chemical analysis laboratory [Southern Scientific Laboratories, Kerry, Ireland] to provide measurements of N, P, K, Mg and Ca content (as % dwt).

To gain some insight into how HF and biochar might affect the photosynthetic capacity of cereal seedlings, a SPAD meter [Konica Minolta SPAD-502 Plus] was used to provide an estimate of leaf chlorophyll levels one week before harvest, with three readings taken in the middle of the largest leaf on each plant and the median value recorded. SPAD readings were taken on a selection of plants from the assays described above: specifically to assess how HF and the form of biochar (Section 2.4 above) and how biochar application rate and HF application rate (Section 2.5 above) influenced SPAD levels.

### 2.7. Statistical Analysis

Data were collated and graphics created using Microsoft Excel. All statistical analyses were performed using Genstat v21 (VSN International Ltd., Hemel Hempstead, UK). When different plant species were used in trials, the data for each species were analyzed separately. In most cases, shoot dry weight data were analyzed using analysis of variance (ANOVA), with approximations to normality of errors and equality of variances checked visually [43]. Generally, these ANOVA were straightforward, and involved two explanatory factors along with an interaction term.

For the trial examining the effects of the different biochar feedstocks on the growth of barley (see Section 2.4) a slightly more complicated explanatory model was required, because assigning replicates to biochar form (powdered or granular) was not possible in the treatments where no biochar was applied. For this trial, the data for the different biochar feedstocks were analyzed separately, and the ANOVA model consisted of HF as a two-level fixed factor and then biochar form (two levels) nested within biochar application rate (three levels). 

For the examination of HF application rate on shoot dry weight, polynomial regression curves were fitted for each response variable using a quadratic model of the form:

Shoot dwt = *a* + *b*(HF) + *c*(HF^2^).

where *a, b*, and *c* are constants, and HF is the application rate in g. For these polynomial relationships, HF_Max_, which is the HF application rate (per pot) that would produce the maximum mean shoot dry weight under these conditions, can be estimated using the formula −*b*/2*c*.

In the examination of the effects of HF and biochar on foliage chemical content, linear models were used with the HF application rate set as a numerical explanatory variable and the presence of biochar set as a fixed categorical factor.

## 3. Results

### 3.1. The Effect of HexaFrass™ on Cereal Shoot Growth: Application Rate and Basal Nutrient Conditions

**Figure 1:**
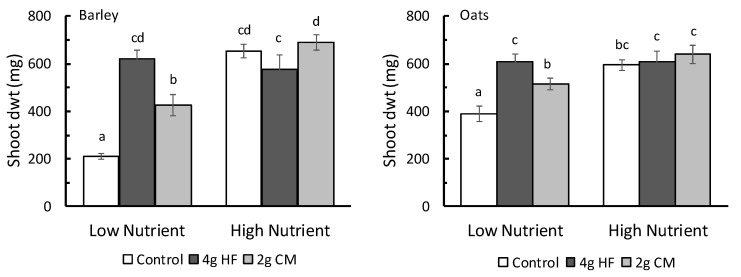
The response of barley and oat shoot dry weight (dwt; mg; mean ± se; N = 10) grown under greenhouse conditions to the application of HexaFrass (HF; 4 g/pot) and chicken manure organic fertilizer (CM; 2 g/pot) with low nutrient and high nutrient potting media. Treatments not sharing the same letter code are separated by Fisher’s LSD (*p* < 0.05).

**Figure 2:**
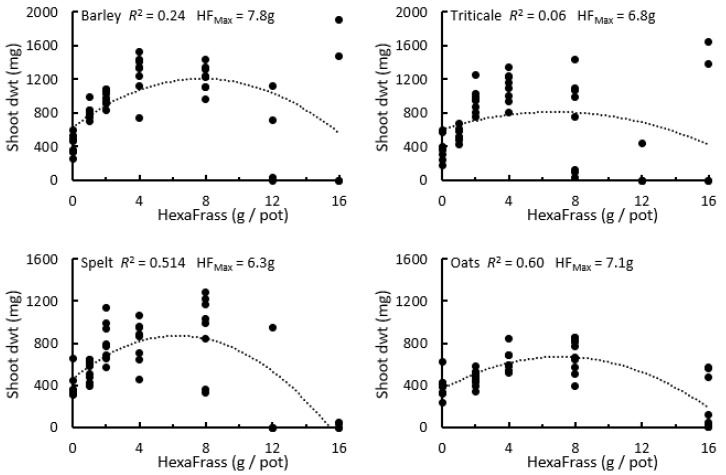
The relationships between shoot dry weight (dwt) of barley, triticale, spelt, and oats with application rate of HexaFrass (g/pot). Plants that did not survive until harvest were assigned a shoot dry weight of zero. HF_Max_ is the application rate producing the predicted maximum shoot dwt.

When examining how basal nutrient levels affected the response of plants to HF, for both barley and oats, there was a significant statistical interaction (F_2_,_54_ > 5, *p* < 0.01) between fertilizer treatment and the potting mix treatment (Figure 1). As expected, in the absence of any fertilizers both plants gained more shoot dry matter in the high nutrient potting mix compared with the low nutrient mix, although this effect was more pronounced in barley (≈3-fold increase) than in oats (≈1.5-fold increase, see Figure 2). In the low nutrient potting media, the application of 2 g of CM and, especially, 4 g of HF produced an increase in barley and oat shoot dwt compared with that achieved in the respective no-fertilizer treatments. In the high nutrient potting mix, however, neither the HF nor the CM resulted in higher shoot dwt compared with that obtained in the controls for either plant species (Figure 1).

In the low nutrient potting medium, the shoot dwt of all four cereals exhibited non-linear relationships with HF application rate (Figure 2). For spelt (*R*^2^ = 0.51) and oats (*R*^2^ = 0.60) these relationships were well described by quadratic functions. For barley (*R*^2^ = 0.24) and triticale (*R*^2^ = 0.06), however, these patterns were not so clear, largely because half of plants in the 16 g HF treatment did not survive, but the surviving plants still produced very high shoot dry weights (Figure 2). Based on the fitted polynomial curves, the estimated optimal HF application rates in this experimental system were all between 6 to 8 g per pot: 6.3 g/pot for spelt, 6.8 g/pot for triticale, 7.1 g/pot for oats, and 7.8 g/pot for barley. For the cereals where shoot dry matter (DM; %) was examined (barley, triticale, spelt), there were non-linear negative relationships between the HF application rate and the DM content (Supplementary Figure S1).

### 3.2. Response of Four Cereals to a Combination of HexaFrass™ and Biochar

**Figure 3:**
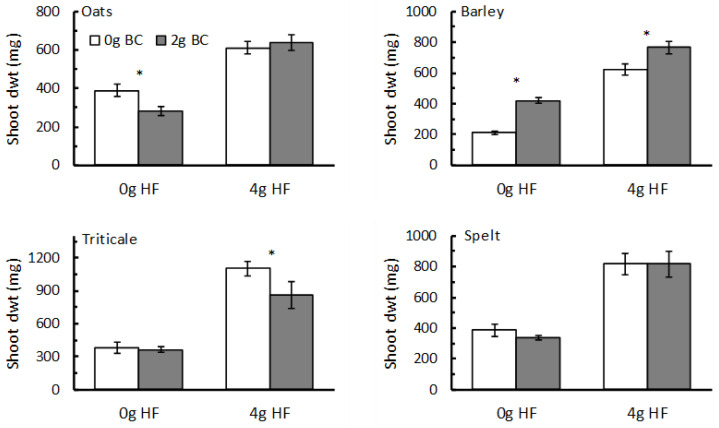
The response of oats, barley, triticale, and spelt (shoot dry weight; dwt; mg; mean ± se; N = 8) to addition of HexaFrass (HF; 0 g or 4 g/pot) and biochar (BC; 0 g/pot—white columns; 2 g/pot—dark columns)). *—significant difference between biochar treatments for the same HF level as indicated by Fisher’s LSD (*p* < 0.05).

In the trials investigating how HF and biochar might interact in their effects on plant growth, adding 4 g of HF per pot had highly significant effects (F_1,28_ > 60; *p* < 0.001) on the shoot dwt of all four cereal species (Figure 3). The effects of adding 2 g of biochar, however, were not consistent among the four plants. For spelt, there was no effect of biochar on shoot growth (F_1,28_ = 0.20; *p* = 0.659), whereas for barley, biochar significantly increased shoot growth overall (F_1,28_ = 37.1; *p* < 0.001), and this effect was seen in plants that had received 0 g of HF and those that had received 4 g of HF (Figure 3). In oats, there was a significant interaction between HF and biochar (F_1,28_ = 4.15; *p* = 0.049), with biochar having no effect on shoot growth when 4 g HF was added, but causing a reduction in growth when no HF was applied. Triticale showed no overall significant response to biochar (F_1,28_ = 2.91; *p* = 0.099), but the LSD value indicated that there was a significant reduction in shoot growth when biochar was added to plants that had also received 4 g HF (Figure 3). 

### 3.3. Response of Barley to Four Types of Biochar in Granular and Powdered Forms

**Figure 4:**
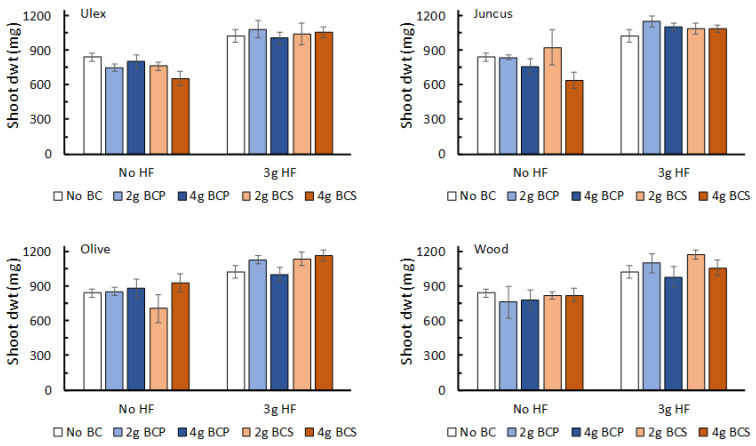
The response of barley (shoot dry weight, dwt; mean ± se; N = 8 per treatment) grown under greenhouse conditions to the addition of biochar created from four different feedstocks: *Ulex* sp., *Juncus* sp., olive stones and hardwood, and wood chips. Biochar was added to pots at three rates (0 g, 2 g, or 4 g per pot) and was added in two forms, powdered (BCP) and ‘sieved’ (BCS), with grains between 3–7 mm. These biochar treatments were combined with the addition of HexaFrass at two rates (0 g and 3 g per pot) to produce ten treatments in total for each biochar.

In these trials, the overriding result was that the addition of 3 g HF to the growing media resulted in a significant increase in barley shoot dwt (F_1,56_ > 60, *p* < 0.001; Supplementary Table S2), regardless of the biochar feedstock, quantity, and form (Figure 4). For the biochar based on *Ulex*, spruce, and olive stones, there were no effects of adding 2 g or 4 g of biochar, or adding powdered or granular forms, on barley shoot dwt (Supplementary Table S2). For the biochar based on *Juncus*, there were significant differences in shoot dwt depending on the amount of biochar added (F_1,56_ > 3.35, *p* = 0.042), although these differences were not easily explained, as the average shoot dwt when 2 g of biochar was applied was significantly greater than that when 4 g of biochar was applied, with the no-biochar treatment producing intermediate shoot dwt (Figure 4). 

### 3.4. Response of Barley and Oats to Biochar Application Rate with and without HexaFrass™

**Figure 5:**
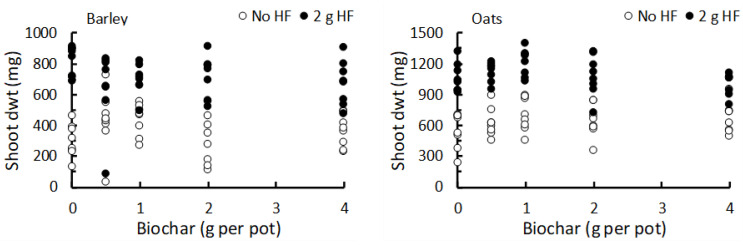
The response of barley and oats (shoot dry weight, dwt; mean ± se; N = 8 per treatment) grown under greenhouse conditions to different application rates of powdered biochar created from olive stones and hardwood feedstock. The biochar treatments were combined with the addition of HexaFrass at two rates (0 g and 2 g per pot) to produce ten treatments in total for each plant species.

In these trials, both barley (F_1,70_ = 182.4, *p* < 0.001) and oats (F_1,70_ = 192.0, *p* < 0.001) produced significantly higher shoot growth following the addition of 2 g of HF per pot, but there were no significant differences among the five application rates of biochar (barley: F_4,70_ = 1.76, *p* = 0.147; oats: F_4,70_ = 2.33, *p* = 0.064; Figure 5). For oats, the interaction between the biochar application rate and the addition of HF was also statistically significant (F_4,70_ = 3.90, *p* = 0.006). The reasons behind this significant interaction are not totally clear, but seem to arise from the 1 g biochar application rate producing the highest shoot growth both with and without the addition of HF, whereas the lowest growth without HF occurred in the 0 g biochar rate, and the lowest growth with HF occurred in the 4 g biochar application rate. 

### 3.5. Qualitative Changes in Cereal Foliage Following Application of Biochar and HexaFrass™

**Figure 6:**
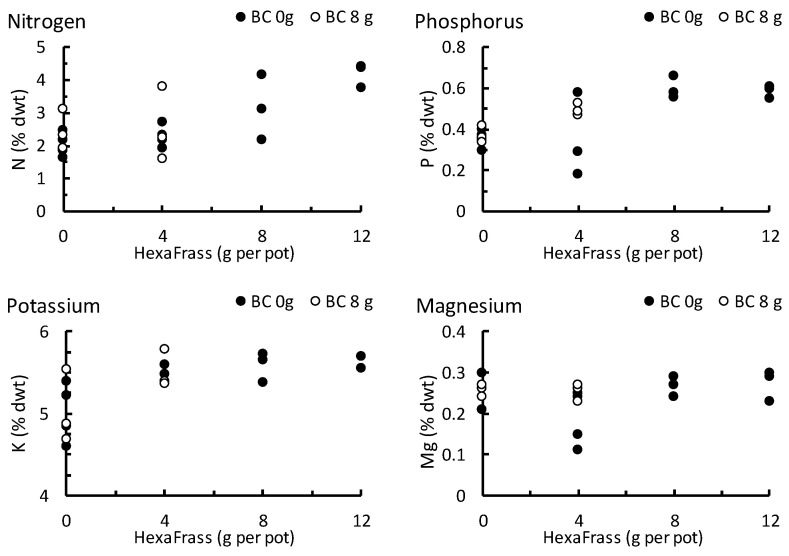
The chemical content (% dwt) of barley foliage grown under greenhouse conditions in response to different application rates (g per pot) of HexaFrass and olive stone biochar (BC). Biochar was only applied for the 0 g and 4 g HexaFrass treatments.

In the trial growing barley in large (2 L) pots and examining the effect of HF and biochar on foliage quality, the application of HF caused an increase in shoot fwt and shoot dwt, and a decrease in DM (%) content. There was no effect of biochar application on shoot growth (Supplementary Table S3). In terms of foliage chemistry, the HF application rate was positively related to shoot nitrogen (*p* < 0.001), phosphorous (*p* < 0.001), and potassium (*p* = 0.003) content, but not magnesium (*p* = 0.487; Figure 6). The application of biochar, and the interaction between biochar and HF, did not affect any of the foliage nutrients measured (*p* > 0.2 in all cases). 

**Figure 7:**
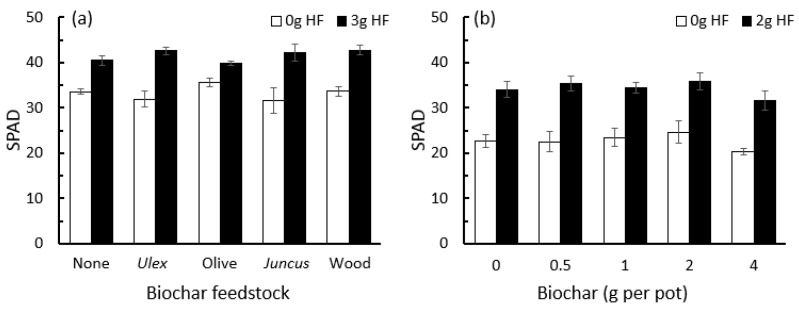
SPAD readings (mean ± se) for barley foliage grown under greenhouse conditions in response to application of HexaFrass (HF) and (**a**) biochar created from different feedstocks and (**b**) olive stone biochar applied at different rates (g per pot).

In the trial examining the effect of HF and powdered biochar from different feedstocks on SPAD levels of barley foliage, the HF caused a significant (25%) increase in SPAD levels (F_1,56_ = 97.9, *p* < 0.001), whereas there was no effect of any of the biochars (F_4,56_ = 1.06, *p* = 0.386; Figure 7a). In the trial investigating the effect of the biochar application rate on the SPAD levels of barley foliage, there was again no relationship between the quantity of biochar added and SPAD readings (F_4,70_ = 1.53, *p* = 0.204), whereas the addition of 2 g of HF to each pot caused a significant (51%) increase in SPAD levels (F_1,70_ = 102, *p* < 0.001; Figure 7b).

## 4. Discussion

The general findings of the multiple trials performed in this investigation are that HexaFrass (HF), an insect frass fertilizer (IFF) produced from black soldier flies, can enhance seedling shoot growth in several species of cereals. The magnitude of the positive effect of HF on shoot growth was similar to that achieved with a recognized organic soil amendment, chicken manure pellets, which is a result that has also been observed in previous experiments using the same greenhouse experimental system [24,35]. The positive effects of applying HF only occurred when basal soil nutrients were low, and only when the HF is not applied in excessive amounts, suggesting the benefits of HF might be conditional on the plants experiencing some form of nutrient deficit [24,35]. 

Previous studies have also indicated that plants do not respond well to excessive amounts of IFF [44,45], and, in the current study, the shoot yield per pot for all four cereals exhibited non-linear functions with HF application rate. Although these relationships did not follow quadratic functions in every case, similar non-linear functions relating shoot dry weight and IFF application rate have been observed for chicory and plantain [35], as well as for basil, parsley, and lettuce seedlings [24]. 

The low shoot yield observed at the highest applied HF rate (16 g per pot) was, with the exception of oats, primarily due to 50% of seedlings not surviving, which was similar to that observed for chicory and plantain [35]. Non-linear relationships between plant growth and applied N are common and have been described previously for cereals such as wheat, although these relationships can be of slightly different profiles when N is applied as different forms (e.g., urea, nitrate, ammonium, glutamine; [46]). As the N content of HF is only ≈3–4%, it seems unlikely that the negative impacts of the high HF application rates were due to excessive N, and it is possible that the very young plants responded to other bioactive substances in the IFF (e.g., chitin) or the creation of an excessively organically rich growing medium. For some of the cereals in this study, especially barley and triticale, although only 50% of the plants survived at the highest HF application rate, the survivors subsequently performed well and accumulated the highest shoot dry weights. It is possible that once seedlings become established they can cope with—and then benefit from—higher quantities of IFF. Future research might investigate the above hypotheses by applying IFF to seedlings of different ages or different development stages, in addition to comparing how plants respond to nutrients supplied as IFF with plants that are supplied nutrients directly in mineral form.

By supplying biochar in different particle sizes (finely ground versus coarsely crushed), from four different feedstocks (*Juncus*, *Ulex*, olive stones, soft wood), and at different application rates, we had expected to identify at least some combinations where plant performance was modified. Nevertheless, overall, the application of biochar from different feedstocks, in different forms, or at different application rates, did not have any consistent positive or negative effects on shoot dry weight. Although in one trial, the addition of biochar to the growing medium increased the shoot dry weight of barley, in the same trial biochar caused a decrease in the shoot weight of triticale. Additionally, the positive effect of biochar on barley was not apparent in the other trials we performed. Our results with respect to biochar are similar to several previous reports where biochar application had no significant effect on above ground biomass or yield of cereals such as maize and sorghum [21,40] and spring barley [39]. We also found no evidence of any consistent synergistic effects of biochar and HF on plant performance [40], a phenomenon which has been shown to occur in many prior investigations [35,39,47,48]. It is possible that many of the pathways through which biochar can facilitate plants were not present in our experimental system. For example, the plants did not experience any drought stress because they were watered regularly, and the potting mixes used were broadly neutral (pH 6–7) so that no liming effect could occur. Additionally, because all of our plants were grown on individual trays to retain nutrients, the addition of biochar may not have been able to facilitate plants by promoting nutrient retention or nutrient availability. Nevertheless, the same experimental system was able to clearly demonstrate the positive effects of applying HF (and chicken manure fertilizer), and in some cases it must be accepted that commonly used organic amendments, such as biochar and those based on seaweed and humic substances, do not always produce the positive effects on plant growth that might be expected [49,50,51]. 

The addition of HF produced increases in foliage NPK in barley, which suggests that the applied HF was providing sufficient nutrients to not only increase shoot dry weight but to also increase the levels of macronutrients within the plant [52]. Shoot N of cereals will generally increase with the amount of applied N [46], and future work would benefit from comparing the foliage nutrient levels obtained with mineral fertilizers with those obtained when applying HF as a raw product or as an aqueous extract containing only the soluble nutrients. Although not a primary aim of this investigation, the higher nutrient levels in dried shoots could have additional benefits in terms of increased nutritive value of barley or oat straw used as a component of livestock rations [53].

The application of HF significantly increased SPAD values, which is in agreement with previous reports where IFF produced SPAD values at least equivalent to NPK fertilizers and organic composts [54,55]. Chlorophyll production, and thus photosynthetic activity, can be heavily limited by soil nitrogen availability, and the SPAD results reported here reinforce that the beneficial effects of IFF in our trials likely arose because of enhanced macronutrient supply [56]. 

In our trials the application of biochar at different rates or from different feedstocks did not affect foliage nutrient levels or SPAD values. From the literature, the effect of biochar on SPAD values varies considerably: for example, in wheat, Ali et al. (2019) reported an increase in wheat leaf chlorophyll when biochar was coapplied with farmyard manure [47], while Sun et al. (2019) observed that biochar had no effect on wheat leaf chlorophyll [57]. Conversely, Asai et al. (2009) found that biochar significantly reduced the SPAD of rice at one site but not at another, which was attributed to biochar reducing plant N uptake that was caused by the creation of a high soil C/N ratio [58]. This latter hypothesis is supported by data from Huang et al. (2019) who found that basil plants grown in a biochar-based growing media had lower SPAD values than those grown in a peat-based control [59]. Based on these ideas, the lack of a SPAD response in our study may have transpired because the relatively small amounts of biochar we added were not sufficient to substantially affect the C/N ratio of the growing medium and/or reduce N uptake. 

As described above, we concede that, although we have performed multiple trials and identified some consistent effects, there remain several limitations to our investigations. For example, plants were grown in small containers under benevolent greenhouse conditions, and grown only for a short time which meant that plants did not flower and/or produce grains. Nevertheless, the evaluation of different IFFs on different crop species is still a relatively recent discipline, and short-term trials such as these provide a rapid and cost-effective screening process to identify consistent patterns and situations where variability occurs [37,60]. Future studies should aim to examine the effects of IFF and biochar on cereals over longer periods, and to full grain yield, when the implications of low nutrient availability may become more apparent.

## 5. Conclusions

Overall, the results reported here indicate that application of HF, a commercial IFF produced from black soldier flies in Ireland, can increase the shoot dry matter of four cereal species, and also increase foliage nutrient and chlorophyll levels. These results indicate that HF offers good potential as an organically acceptable soil amendment for low-input, organic, or ecologically guided cereal production systems. We found no evidence that the application of biochar increased shoot growth or modified foliage quality. Nevertheless, both insect frass fertilizers and biochar should be investigated further to look at longer-term effects on plant performance and, ultimately, cereal yield and quality. Additionally, although biochar did not enhance plant performance, there was equally no indication of any negative effects, which suggests that the application of biochar to farm soils may still have value in terms of carbon storage and reducing whole-farm carbon budgets.

## Data Availability

Data are available upon request from the corresponding author.

## Supplementary Materials

The following supporting information can be downloaded at: https://www.mdpi.com/article/10.3390/plants12051071/s1, Supplementary Tables and Figure can be found in the Supplementary Materials.
