# Supplementary material for: The Effects of Two Organic Soil Amendments, Biochar and Insect Frass Fertilizer, on Shoot Growth of Cereal Seedlings
Source: Plants (Basel). 2023 Feb 27;12(5):1071. doi: 10.3390/plants12051071 (PMC10004817; doi:10.3390/plants12051071)
Supplement: Supplementary file 1 [file plants-12-01071-s001.zip › plants-2140042-supplementary.pdf]

Supplementary Document S1

Summary of trials performed investigating response of cereals to application of HexaFrass (HF) insect frass fertilizer and biochar, with section of Results where results are described and summary of main findings.

Results Section	Plants	Potting mix	HexaFrass (g per pot)	Biochar (g per pot)	Sample size	Main Results
3.1	Barley<br>Oats	Low & High	4	n/a	19	HF increased shoot dwt<br>HF effect greater than that obtained with 2 g chicken manure<br>HF effect reduced when plants grown in high nutrient potting mix
3.1	Barley<br>Oats<br>Triticale<br>Spelt	Low	1 - 16	n/a	4 - 10	Shoot growth exhibits non-linear relation with HF application rate<br>Small quantities of HF increase shoot dwt for all cereals<br>Large quantities of HF are applied positive effect diminishes<br>Large quantities can cause mortality of seedlings
3.2	Barley<br>Oats<br>Triticale<br>Spelt	Low	4	2	8	HF consistently increased shoot dwt<br>No consistent effects of adding biochar
3.3	Barley	Low	3	2 and 4<br>4 x feedstocks<br>fine powder<br>coarse sieved	6 - 12	HF consistently increased shoot dwt<br>No consistent effect of biochar quantity on shoot dwt<br>No consistent effect of biochar feedstock on shoot dwt<br>No consistent effect of biochar particle size on shoot dwt
3.4	Barley<br>Oats	Low	2	0.5 - 4	8	HF consistently increased shoot dwt<br>No relationship between shhot dwt and biochar application rate
3.5	Barley	Low	0 - 12	8	3 - 4	HF increased shoot N, P, K but not Mg<br>Biochar had no effect on foliage chemistry
3.5	Barley	Low	3	4<br>4 x feedstocks	6 - 12	HF increased foliage chlorophyll content (SPAD)<br>Chlorophyll content not affected by biochar application rate<br>Chlorophyll content not influenced by biochar feedstock
3.5	Barley	Low	2	0.5 - 4	8	HF increased foliage chlorophyll content (SPAD)<br>Chlorophyll content not affected by biochar application rate

Supplementary Document S2. Response of shoot dry matter content (dm; %) of cereals to addition of HexaFrass fertilizer at different rates.

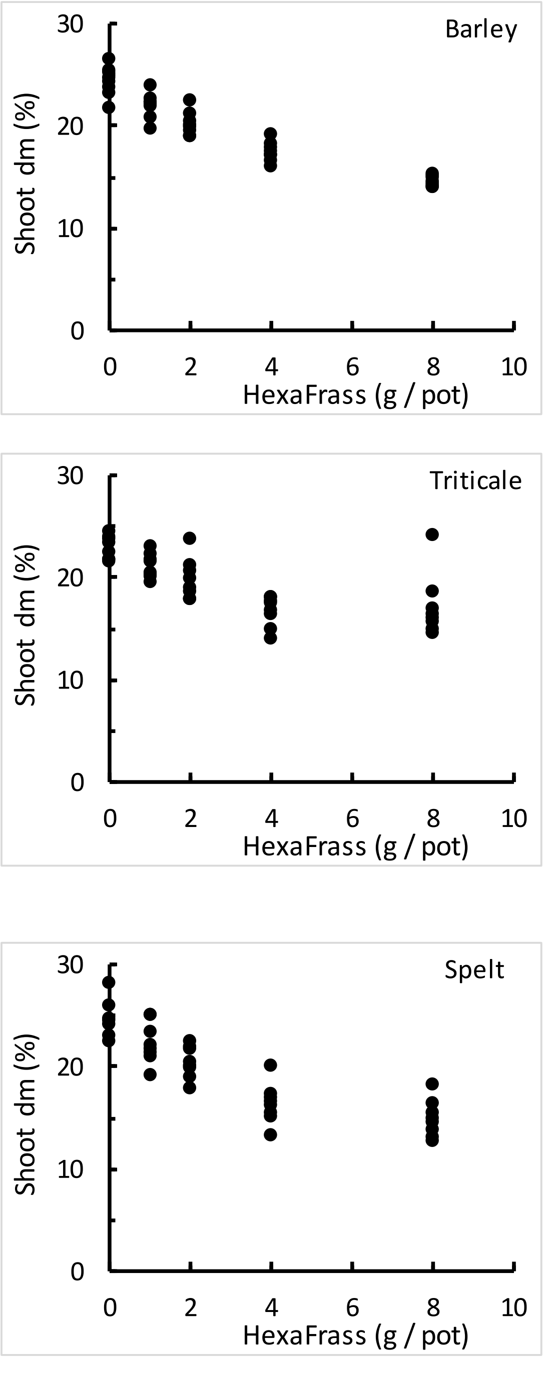

Supplementary Document S3.

Results of ANOVA (P-values) assessing the effects of 3 g HF (**HF**), amount of biochar (0, 2 4 g; **BCg**), and form of biochar (powder/grains; **BCForm**) on growth of barley shoots (dry weight). Data for biochar created using different feedstocks (*Ulex*, olive stones & hardwood, *Juncus*, soft wood/spruce) were analyzed separately.

Analysis was performed using nested ANOVA model used: dwt ~ HF * (BCg/BCForm)

Factor	df	Ulex	Olive	Juncus	Spruce
HF	1	<.001	<.001	<.001	<.001
BCg	2	0.326	0.555	0.042	0.599
HF × BCg	2	0.351	0.14	0.074	0.324
BCg × BCForm	2	0.693	0.169	0.469	0.497
HF × BCg × BCForm	2	0.212	0.364	0.508	0.962
Residual	56				
Total	65				

Supplementary Document S4

Shoot fresh weight (Fwt; g), dry weight (Dwt; g) and dry matter content (DM; %) of barley plants grown in 2 L pots under glasshouse conditions, with the addition of HexaFrass (HF) and Biochar (BC). Values are mean ± SE.

Treatment	N	Fwt (g)	Dwt (g)	DM (%)
Control	8	18.7 ± 2.0	2.5 ± 0.2	13.8 ± 0.6
BC 8g	6	14.7 ± 2.0	2.1 ± 0.2	14.3 ± 0.9
HF 4g	8	26.1 ± 0.9	3.1 ± 0.1	11.7 ± 0.4
HF 4g + BC 8g	6	26.2 ± 1.0	3.1 ± 0.1	12.1 ± 0.4
HF 8g	6	34.1 ± 2.0	3.8 ± 0.3	11.1 ± 0.3
HF 12g	6	39.2 ± 2.9	3.9 ± 0.4	9.9 ± 0.4
